# Effects of ambroxol on the autophagy-lysosome pathway and mitochondria in primary cortical neurons

**DOI:** 10.1038/s41598-018-19479-8

**Published:** 2018-01-23

**Authors:** J. Magalhaes, M. E. Gegg, A. Migdalska-Richards, A. H. Schapira

**Affiliations:** Department of Clinical Neuroscience, Institute of Neurology, University College London, London, NW3 2PF UK

## Abstract

Glucocerebrosidase (*GBA1*) mutations are the major genetic risk factor for Parkinson’s Disease (PD). The pathogenic mechanism is still unclear, but alterations in lysosomal-autophagy processes are implicated due to reduction of mutated glucocerebrosidase (GCase) in lysosomes. Wild-type GCase activity is also decreased in sporadic PD brains. Small molecule chaperones that increase lysosomal GCase activity have potential to be disease-modifying therapies for *GBA1-*associated and sporadic PD. Therefore we have used mouse cortical neurons to explore the effects of the chaperone ambroxol. This chaperone increased wild-type GCase mRNA, protein levels and activity, as well as increasing other lysosomal enzymes and LIMP2, the GCase transporter. Transcription factor EB (TFEB), the master regulator of the CLEAR pathway involved in lysosomal biogenesis was also increased upon ambroxol treatment. Moreover, we found macroautophagy flux blocked and exocytosis increased in neurons treated with ambroxol. We suggest that ambroxol is blocking autophagy and driving cargo towards the secretory pathway. Mitochondria content was also found to be increased by ambroxol via peroxisome proliferator-activated receptor gamma coactivator 1-alpha (PGC1-α). Our data suggest that ambroxol, besides being a GCase chaperone, also acts on other pathways, such as mitochondria, lysosomal biogenesis, and the secretory pathway.

## Introduction

Glucocerebrosidase (GCase) is the enzyme responsible for breaking down glucocerebroside into glucose and ceramide in the lysosome. Homozygous mutations in the *GBA1* gene, which encodes GCase, causes deficiency of this enzyme and consequently accumulation of glucocerebroside in the lysosomes, causing Gaucher’s Disease (GD)^1^. GD is a rare autosomal recessive lysosomal storage disease that has been associated with synucleinopathies, including Parkinson’s Disease (PD)^2,3^. Heterozygous or homozygous *GBA1* mutations are the most important risk factor for PD and are found in 7–15% of PD subjects, and 25% of those with Ashkenazi descent. *GBA1* mutations result in a phenotype very similar to sporadic PD, although PD patients carrying *GBA1* mutations have earlier onset of PD symptoms and more frequent cognitive impairment^4,5^.

PD is characterized by the presence of α-synuclein - positive inclusions known as Lewy bodies and Lewy neurites in neurons in the substantia nigra, cerebral cortex and hippocampus, and selective loss of dopaminergic neurons in midbrain^6^. The involvement of GCase in the pathology of PD is still not completely understood, however, studies indicate a reciprocal relationship between GCase activity and α-synuclein. Increased α-synuclein levels were shown to decrease lysosomal GCase, whereas, decreased levels of lysosomal GCase leads to accumulation of α-synuclein^7–11^. The lysosome is responsible for degrading lipids, organelles and proteins, such as α-synuclein. Evidence indicates that the lysosomal impairment is an important contributor to the PD pathology^12^.

Other pathways implicated in PD pathology are the Unfolded Protein Response (UPR) and the Endoplasmic Reticulum (ER) stress, both impaired in early stages of PD^13,14^. The role of GCase mutations on ER stress induction have been previously described^15^. GCase is synthesized, folded and translocated to the lysosome to perform its designated function, however, mutant GCase fails to be correctly folded and is arrested in the ER, and then redirected to undergo proteasome degradation via ER associated degradation (ERAD). As a result the levels of GCase in the lysosome are decreased^15^.

Maegawa *et al*. identified ambroxol hydrochloride (ambroxol, Ambx) as a GCase chaperone upon screening the library of US Food and Drug Administration – approved drugs^16^. Ambroxol is an expectorant used in the treatment of respiratory diseases associated with mucus hypersecretion. This drug has the ability to reduce viscosity of secretory products. Besides its mucolytic properties, other effects of ambroxol have been described, such as, being antioxidant, anti-inflammatory and an anaesthetic^17^, and more recently, a potent chaperone for GCase^16^.

Ambroxol treatment has been shown to improve the translocation of mutant GCase to the lysosome, increasing GCase activity in the lysosomes of fibroblast and lymphoblasts carrying *GBA1* mutations^16,18,19^. Notably in control fibroblasts treated with ambroxol, the activity of wild-type (WT) GCase was increased^18–20^. Ambroxol was also found to increase the GCase endogenous activator saposin C and the activity of the lysosomal enzyme Cathepsin D in both control and *GBA1* mutant fibroblasts^18^, with evidence suggesting that ambroxol may activate transcription factor EB (TFEB)^19^, a master regulator of lysosomal biogenesis^21^.

Treatments in fly models that carried *GBA1* mutations and presented a PD phenotype, showed that ambroxol can reverse mutant *GBA1* PD like phenotype^20,22^. More recently, mice have been treated with ambroxol. In that study, mice expressing WT, mutant *GBA1*, or overexpressing human α-synuclein mice had increased GCase activity levels in brain upon treatment. Additionally, in mice overexpressing human α-synuclein, ambroxol treatment decreased α-synuclein protein levels^23^. This observation in α-synuclein mice coupled with decreased GCase activity observed in aged and sporadic PD brains^8,24,25^ support the hypothesis that ambroxol is a potential disease modifying therapy for the treatment of not only PD with *GBA1* mutations and GD, but also sporadic forms of PD.

Therefore, in this study we have investigated whether WT GCase activity can be increased by ambroxol in mouse cortical neurons, and thus likely contribute to the elevation in GCase activity observed in adult mouse brain following chaperone treatment^23^. Given the previous finding in fibroblasts we also investigated whether the expression of other lysosomal proteins was increased, and if this was coincident with activation of TFEB. TFEB has also been associated with activation of peroxisome proliferator-activated receptor gamma coactivator 1-alpha (PGC1-α)^26,27^, therefore the effect of ambroxol on this regulator of mitochondrial protein synthesis was also investigated.

## Results

### Ambroxol modifies lysosomal content and function

The optimum concentration of ambroxol for the treatment of neurons was established by treating primary wild-type mouse cortical neurons with three different doses of ambroxol: 10, 30, and 60 µM for 5 days. Cellular viability was measured with the Live/Dead assay. The 60 µM dose caused cell death (63% decrease in the number of live cells, p = 0.003, Fig SI 1A), while 10 or 30 μM had no significant effect on cell death. Consequently, we excluded the highest dose from further studies. The morphology of the neurons was further analysed by measuring the neurite length upon ambroxol treatment at doses 10 and 30 µM. There was no evidence of significant alterations in neurite length at these two dosages (Fig SI 1B), thus we continued our study treating neurons with 10 and 30 µM of ambroxol for 5 days.

**Figure 1:**
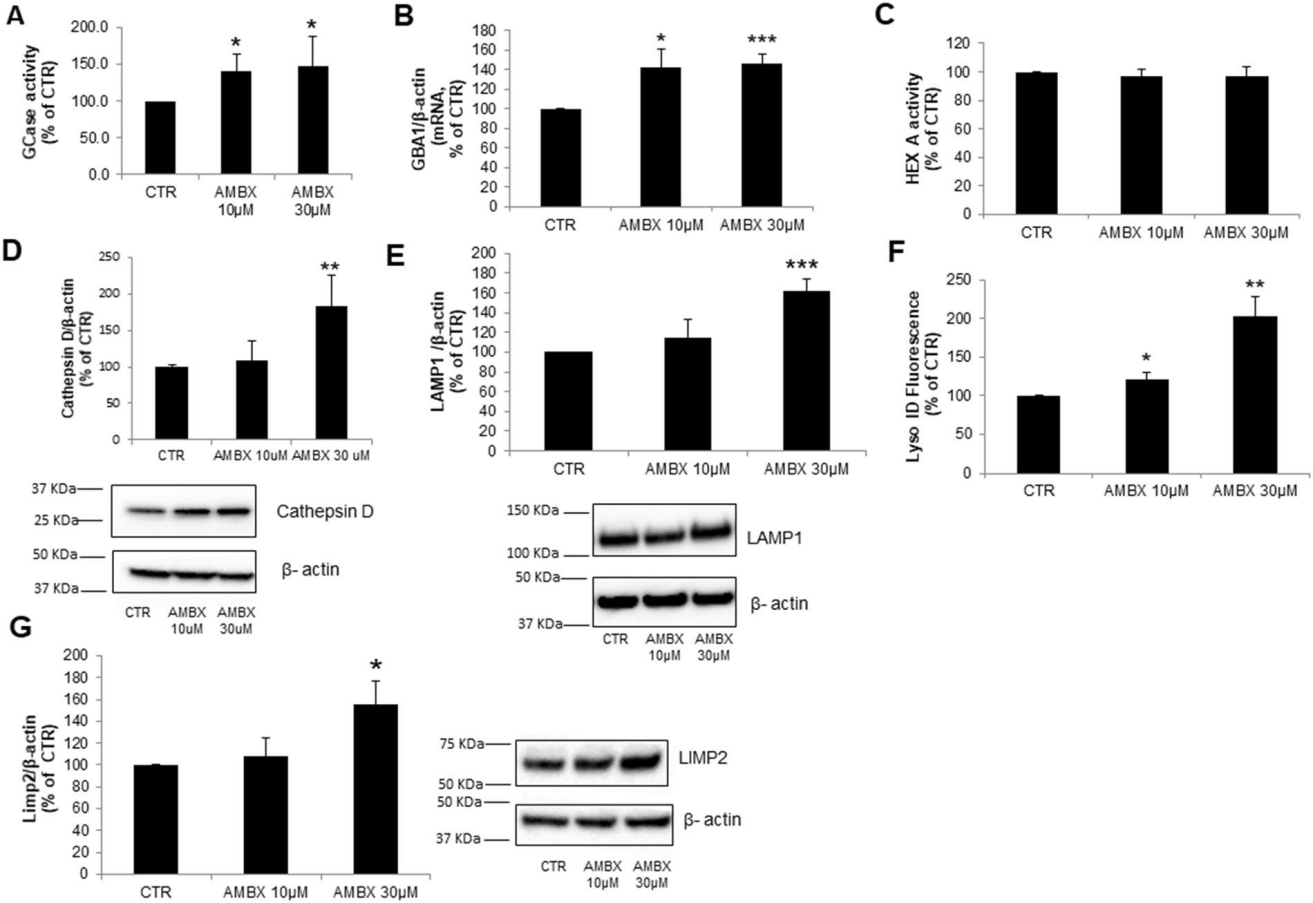
Lysosomal content changes induced by ambroxol. (**A)** GCase activity (n = 4) and (**B**). Gba1 mRNA (n = 3) were increased in neurons treated with 10 µM and 30 µM. (**C**) Hex A activity levels were unchanged upon treatment with 10 µM and 30 µM of ambroxol (n = 4). (**D**) Cathepsin D and (**E)**. Lamp1 protein levels were increased upon treatment with 30 µM of ambroxol compared to control (n = 5 and n = 6 respectively). (**F)** Lyso ID staining showing an increase in acidic vesicles upon treatment with 10 µM and 30 µM ambroxol (n = 4). (**G)** LIMP2 protein levels were increased upon treatment with 30 µM of ambroxol but not with 10 µM (n = 4). Blots have been cropped. Data presented in % of CTR. All data represent mean ± S.E.M. *p < 0.05, **p < 0.01, ***p < 0.001.

Firstly, we looked at the effect of ambroxol on lysosomal content and function. As ambroxol is a GCase chaperone^16^, we started by analysing endogenous wild-type GCase activity upon ambroxol treatment. We detected a similar increase in GCase activity both in the 10 µM (39%, p = 0.05) and 30 µM (47%, p = 0.05) doses (Fig. 1A). To assess if a lower dosage could be used, GCase activity was measured upon treatment with 5 µM of ambroxol, however at this dosage no significant increase in GCase activity was observed, thus we continued our studies using 10 µM and 30 µM dosages.

Levels of *Gba1* mRNA expression were also significantly increased both at 10 µM (42%, p = 0.02) and 30 µM (46%, p = 0.0007) doses (Fig. 1B), which could explain the increase in GCase activity. We then analysed the activity of another lysosomal enzyme, β-hexosaminidase A (HEX A). No differences were observed between control and ambroxol treated cells (Fig. 1C). Mature Cathepsin D (CatD; 28 kDa band) levels were found to be increased at the higher ambroxol concentration (10 µM: no differences; 30 µM: 82% increase, p = 0.003) (Fig. 1D). LAMP1 protein levels were also increased at both doses, however only significantly increased at 30 µM (10 µM: 14% increase, non-significant; 30 µM: 62% increase, p = 0.0006, Fig. 1E). Using the Lyso-ID probe we measured the amount of acidic vesicles such as late endosomes and lysosomes in neurons. Upon treatment with 10 µM of ambroxol the amount of acidic vesicles increased by 20% (p = 0.05) whereas the 30 µM dosage increased the amount by 102% (p = 0.007) compared with control (Fig. 1F). Since LIMP2 is responsible for GCase transport to the lysosome, we analysed its levels upon ambroxol treatment. There were no changes at 10 µM compared to control, however at 30 µM of ambroxol, LIMP2 levels were significantly higher (10 µM: no difference; 30 µM: 56% increase, p = 0.04, Fig. 1G). Altogether the data suggest that ambroxol increases GCase activity and lysosomal content in mouse cortical neurons.

### Ambroxol affects the autophagy pathway

**Figure 2:**
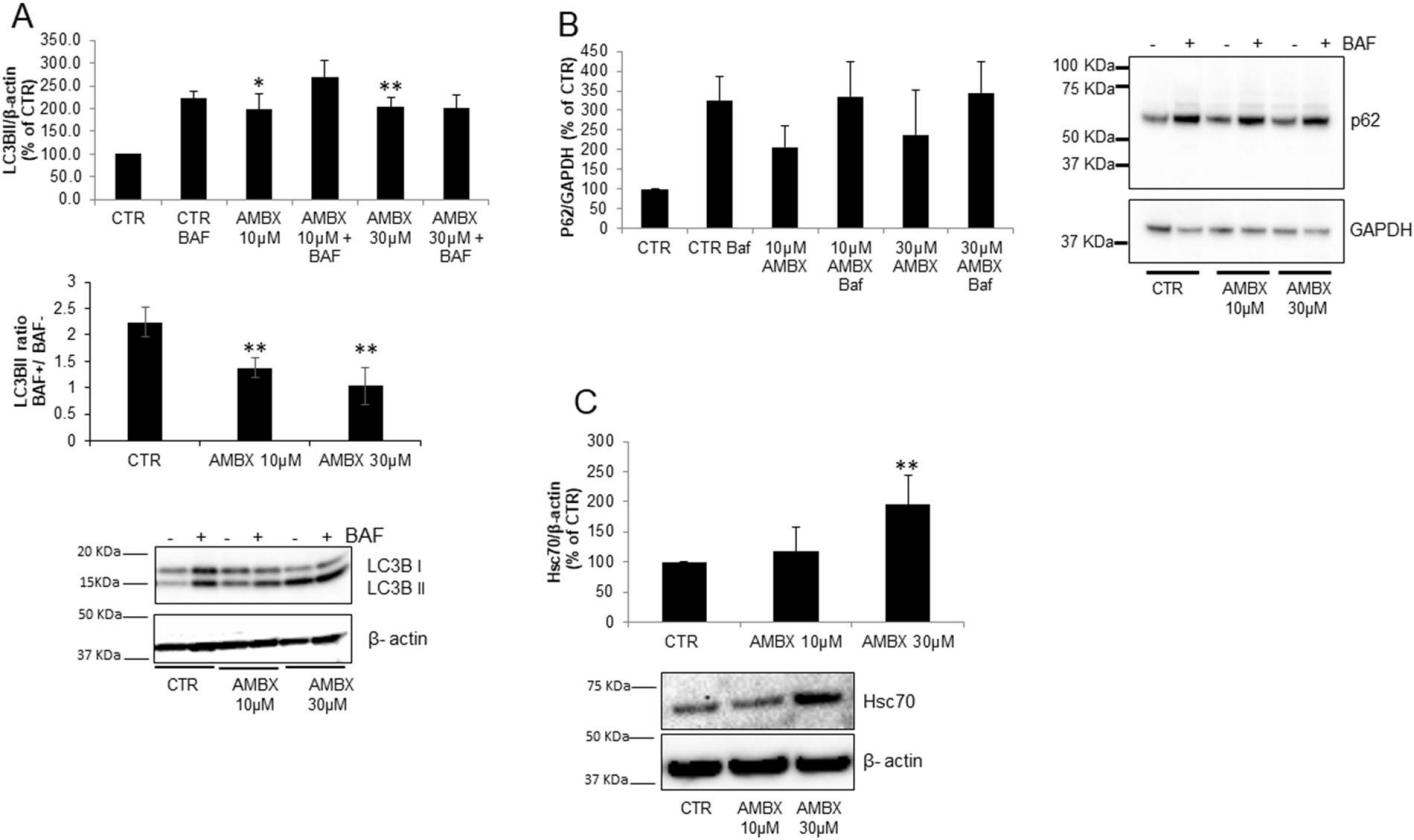
Alteration in autophagy due to ambroxol treatment. (**A)** LC3B-II basal levels increased in ambroxol treated neurons (10 µM and 30 µM) compared to control (n = 3). Upon BAF treatment control neurons (no ambroxol) showed an expected increase in LC3B-II levels whereas in neurons treated with ambroxol, which presented and increase in LC3B-II level, BAF did not induce a change in LC3B-II levels (n = 3). Graphs represent total LC3B-II levels (upper panel) and LC3B-II ratio between BAF + /BAF- samples (middle panel) in each condition. (**B**) P62 levels show a tendency to increase upon treatment with 10 and 30 µM of ambroxol, BAF treatment induces an increase in all conditions (WT, 10 µM and 30 µM) (n = 3). (**C)** Hsc70 levels increased only with 30 µM of ambroxol, as 10 µM did not present changes compared to control neurons (n = 4). Blots have been cropped. Data presented in % of CTR. All data represent mean ± S.E.M. *p < 0.05, **p < 0.01, ***p < 0.001.

To analyse autophagy flux, we detected LC3B-II protein levels in neurons upon ambroxol treatment. We found that ambroxol significantly increased LC3B-II to similar levels at both doses (at 10 µM: 199%, p = 0.02; at 30 µM: 203%, p = 0.003, Fig. 2A). Upon treatment with 100 nM of Bafilomycin A1 (BAF) for 6 h, which increases lysosomal pH and impairs autophagy flux by inhibiting the fusion between the lysosomes and autophagosomes, LC3B-II levels were increased in control cells as expected, and were similar to levels in cells treated with ambroxol alone. However, in ambroxol treated cells, LC3B-II levels were not significantly increased following BAF treatment, compared to just ambroxol treatment alone (Fig. 2A, upper panel). This was also seen when the LC3B-II data was expressed as a ratio of + BAF/-BAF (Fig. 2A, middle panel). This suggests that autophagy flux is blocked by ambroxol. To investigate further, we measured P62/SQSTM1 levels. P62 binds proteins identified for degradation by macroautophagy and helps recruit them to the phagophore, which is then sequestered inside the autophagosome with the cargo and ultimately degraded following fusion with a lysosome. We found that P62 protein levels tended to increase following ambroxol treatment, although this was not significant (Fig. 2B). We also assessed the protein levels of two key mediators of chaperone mediated autophagy, hsc70 and Lamp2a. Lamp2a was unchanged upon ambroxol treatment (data not shown) whereas hsc70 was increased when neurons were treated with 30 µM of ambroxol (10 µM: no differences, 30 µM: 195%, p = 0.008; Fig. 2C).

### Ambroxol activates transcription factor EB (TFEB)

**Figure 3:**
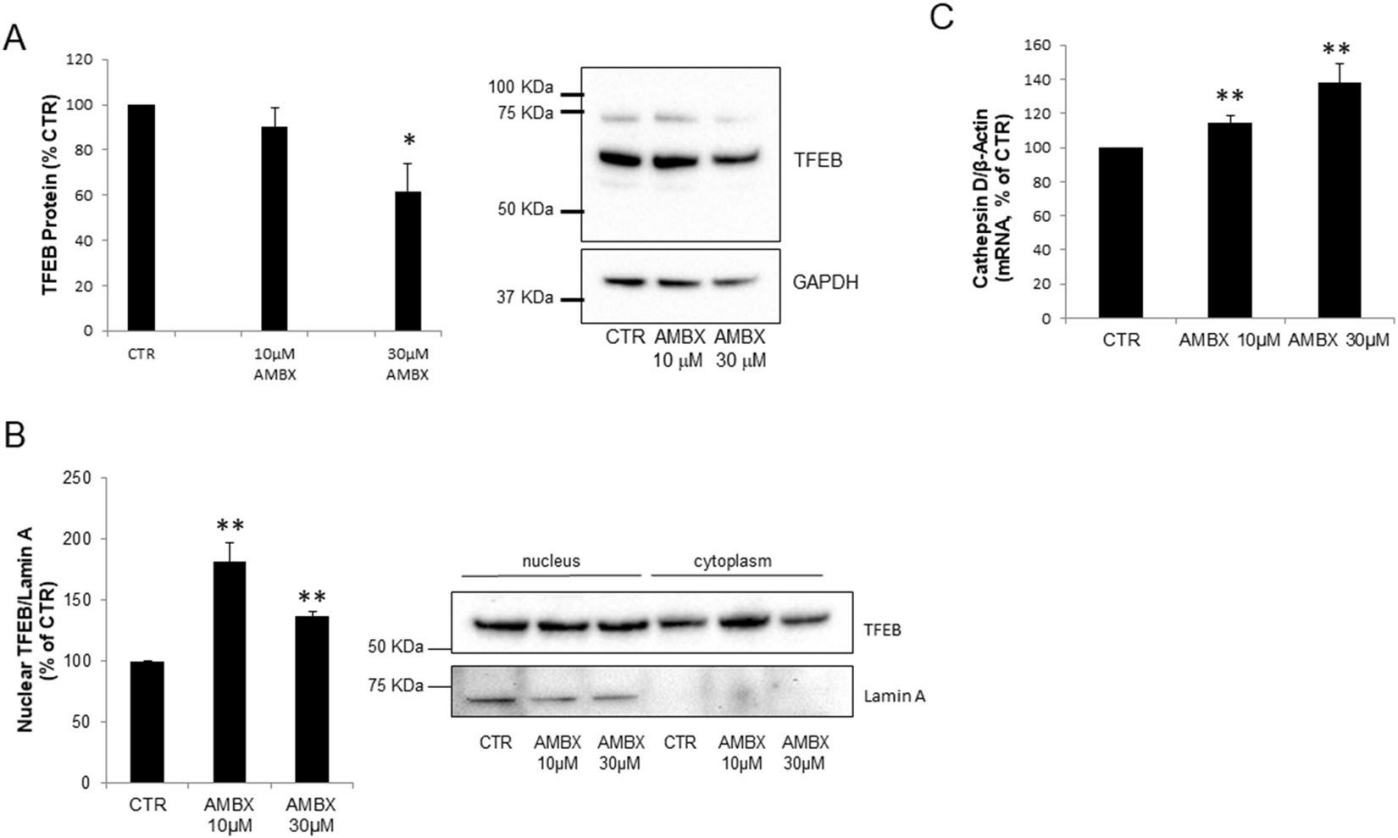
TFEB activation by ambroxol. (**A**) TFEB levels in Triton X-100 cytosolic lysates show a significant decrease in TFEB levels upon treatment with 30 µM of ambroxol treatment and a tendency to decrease with the 10 µM dose (n = 4). (**B)** TFEB translocation to the nucleus was increased upon treatment with both doses of ambroxol (10 µM and 30 µM) (n = 3). Blots have been cropped. **(C**) Cathepsin D mRNA was also increased upon treatment with 10 µM and 30 µM of ambroxol (n = 4). Data presented in % of CTR. All data represent mean ± S.E.M. *p < 0.05, **p < 0.01, ***p < 0.001.

Since we found alterations in lysosomal content and autophagy pathways in neurons treated with ambroxol, and it is well described that TFEB is a master regulator of the lysosomal and autophagy pathway^21,28^, we assessed the activation of TFEB upon ambroxol treatments. TFEB activation is measured by its translocation to the nucleus. We analysed TFEB levels in Triton X-100 cytosolic lysates of neurons treated with ambroxol and detected a decrease in TFEB levels upon treatment (10 µM: 10%, NS; 30 µM: 38%, p < 0.05, Fig. 3A). This might suggest translocation of TFEB to the nucleus. So next we separated cytosolic and nuclear fractions by subcellular fractionation and quantified the amount of TFEB in the nucleus of neurons treated with ambroxol and detected a modest increase in nuclear TFEB both at 10 and 30 µM doses (10 µM: 181%, p = 0.006; 30 µM: 137%, p = 0.01, Fig. 3B). Unfortunately, we were unable to reliably detect endogenous mouse TFEB moving to the nucleus in neurons by immunofluorescence.

We also measured the mRNA levels of Cathepsin D, which is one of the lysosomal genes under the regulation of TFEB, and found it significantly increased at both dosages (10 µM: 114%, p = 0.01; 30 µM: 138%, p = 0.01, Fig. 3C). This finding in conjunction with the increased *Gba1* mRNA levels and lysosomal protein expression (Fig. 1) suggest that ambroxol is activating TFEB.

### Ambroxol effect on mitochondria content

**Figure 4:**
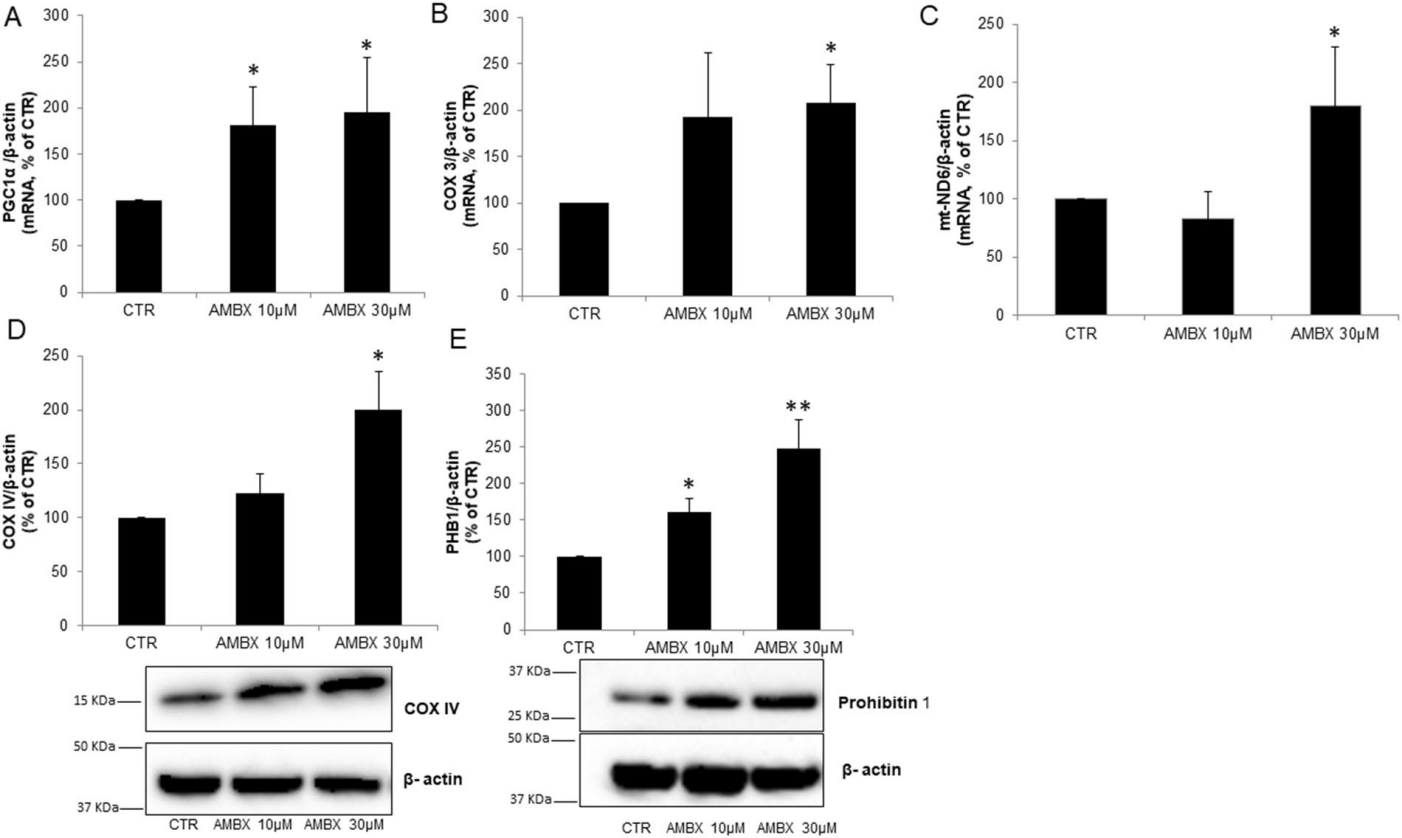
Changes in mitochondria content by ambroxol (**A**). *Pcg1α* mRNA is increased in neurons treated with both 10 µM and 30 µM of ambroxol (n = 3). Both (**B)**. *Cox3* (n = 3) and (**C)**. *mt-Nd6* (n = 4) mRNA were increased with 30 µM of ambroxol but not with 10 µM, compared to control. (**D**) COX IV protein levels were increased with 30 µM of ambroxol compared to control but not with 10 µM of ambroxol (n = 5). (**E)** Prohibitin 1 (PHB1) was increased in both ambroxol treatments, 10 and 30 µM (n = 4). Blots have been cropped. Data presented in % of CTR. All data represent mean ± S.E.M. *p < 0.05, **p < 0.01, ***p < 0.001.

Since TFEB has been shown to be upstream of PGC-1α^26,27^, which among other things is involved in mitochondrial biogenesis^29^, and because mitochondria dysfunction is a feature of PD we analysed PGC-1α mRNA levels. We found PGC-1α mRNA levels increased following treatment of neurons with 10 µM (183%, p = 0.03) and 30 µM ambroxol (195%, p = 0.05) (Fig. 4A). Analysis of mitochondrial DNA encoded COX3 and ND6 mRNA levels showed increased levels after treatment with 30 µM of ambroxol. No changes were observed upon treating with the low dose 10 µM compared to control (COX3: 10 µM: no difference, 30 µM:207%, p = 0.04; ND6: 10 µM: no difference, 30 µM:180%, p = 0.02; Fig. 4B,C). Similarly, COXIV protein levels (nuclear encoded) were increased upon treatment with 30 µM but no differences were observed at 10 µM dose (10 µM: no difference; 30 µM: 200%, p = 0.03; Fig. 4D). The 10 µM dose induced a 60% increase (p = 0.02) whereas the 30 µM dose induced a 147% increase (p = 0.001) in levels of the mitochondrial protein Prohibitin I compared to control neurons (Fig. 4E). Together these results suggest that mitochondrial content and both mitochondrial and nuclear encoded proteins are increased by ambroxol.

### Effect of ambroxol on α-synuclein metabolism

**Figure 5:**
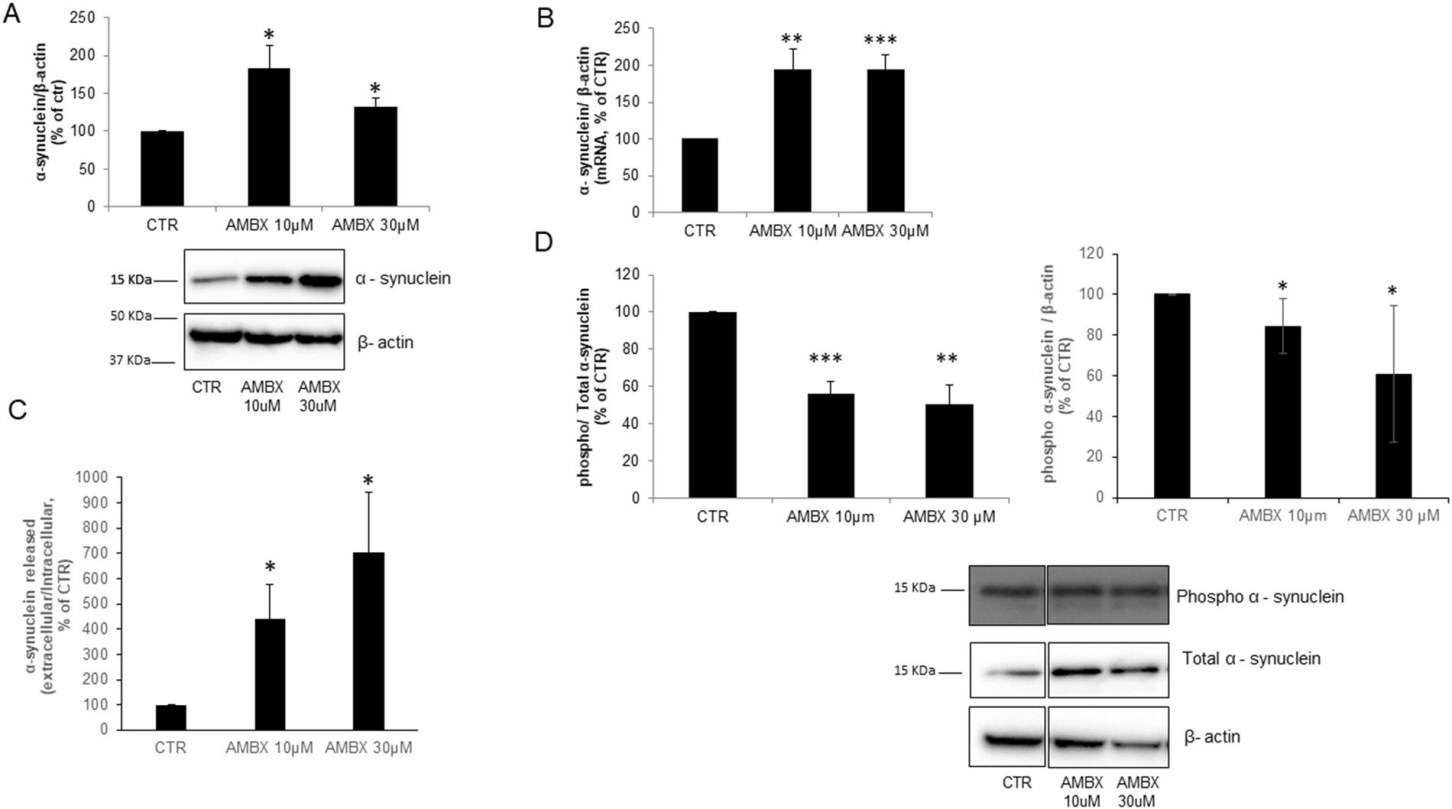
Ambroxol induces changes in α-synuclein metabolism **(A)**. Total α-synuclein increased when neurons are treated with ambroxol 10 µM and 30 µM (n = 4). Blots have been cropped. (**B**) mRNA levels of α-synuclein are also increased in neurons upon treatment with 10 and 30 µM of ambroxol (n = 5). (**C)** Extracellular levels of α-synuclein were measured by ELISA, an increase in release was observed in both 10 and 30 µM of ambroxol (n = 5). (**D**) Phosphorylated (S129) α-synuclein was decreased in neurons with both 10 and 30 µM ambroxol (n = 3). Blot have been cropped from different parts of the same blot, full length blot is shown in Fig SI 3. Data presented in % of CTR. All data represent mean ± S.E.M. *p < 0.05, **p < 0.01, ***p < 0.001.

As a potential therapeutic drug for increasing GCase protein levels in PD, it is important to understand the effects of ambroxol on α-synuclein metabolism. We assessed the levels of total monomeric α-synuclein in neurons upon ambroxol treatment. Both 10 µM and 30 µM ambroxol lead to a significant increase in intracellular total α-synuclein levels (10 µM: 183%, p = 0.02; 30 µM: 132%, p = 0.02, Fig. 5A). This may in part be explained by an increase in α-synuclein mRNA levels (10 µM: 195%, p = 0.004; 30 µM: 193%, p = 0.001, Fig. 5B). No detectable differences in monomeric α-synuclein were observed in urea-SDS fractions following ambroxol treatment (Fig SI 2).

However, we found that the levels of α-synuclein released into cell culture media were significantly increased (10 µM: 440%, p = 0.04; 30 µM: 703%, p = 0.03, Fig. 5C). Although intracellular α-synuclein was increased, intracellular levels of phosphorylated α-synuclein (Ser129; implicated in the pathogenesis of PD) were decreased following treatment with ambroxol (normalized for total α-synuclein: 10 µM: 50%, p = 0.0005; 30 µM: 45%, p = 0.002; normalized for β-actin: 10 µM: 84%, p = 0.03; 30 µM: 61%, p = 0.05 Fig. 5D) compared to control.

### Effect of ambroxol on exocytosis

**Figure 6:**
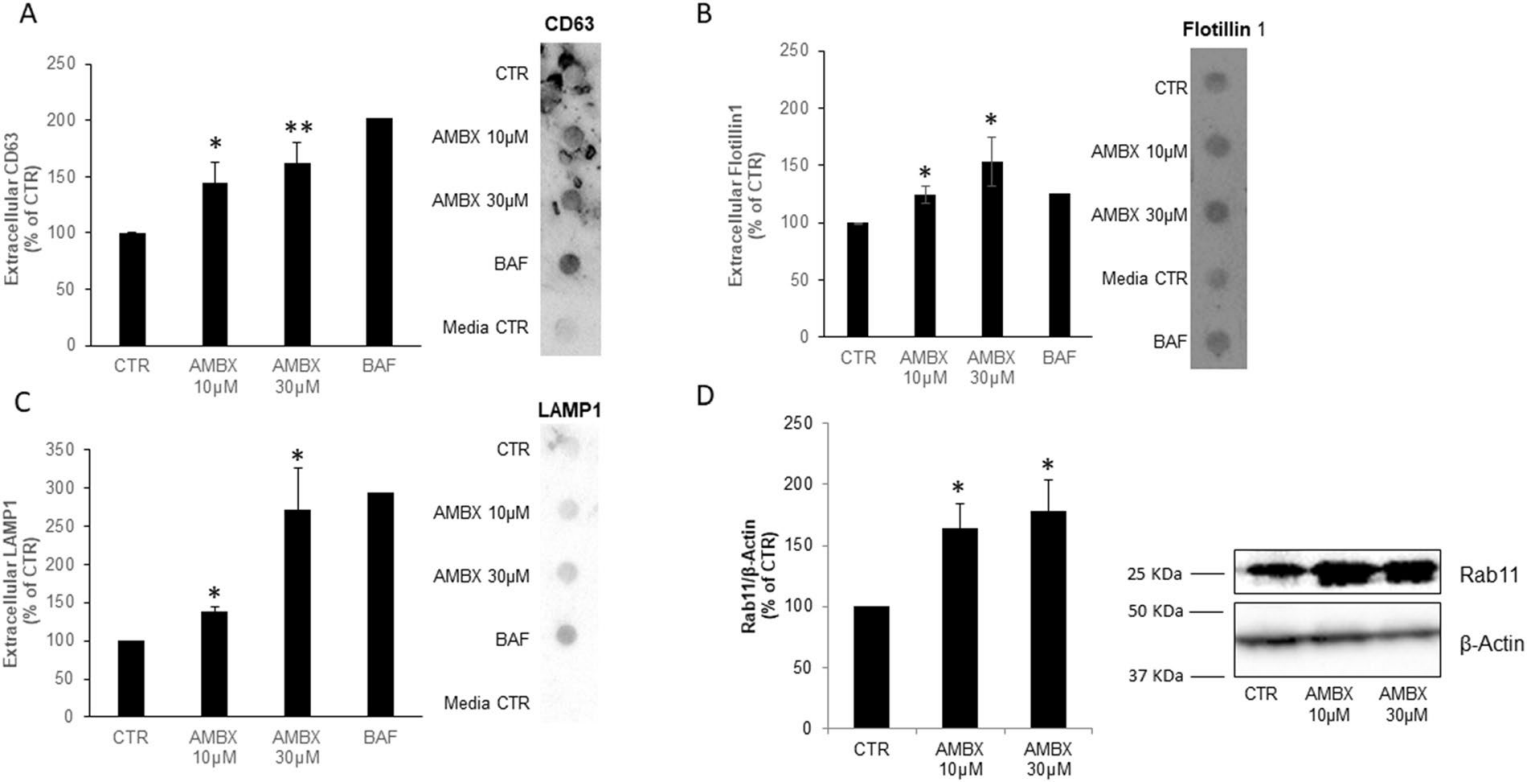
Increased exocytosis upon ambroxol treatment. (**A)** CD63 (n = 5), (**B)** Flotillin 1 (n = 3) and (**C)** Lamp1 (n = 3) release was increased in neurons treatment with 10 or 30 µM of ambroxol. Bafilomycin A1 was used as a positive control of exocytosis. (**D)** Rab 11 intracellular levels were also increased in neurons treated with 10 or 30 µM of ambroxol (n = 4). Data presented in % of CTR. Blots have been cropped. All data represent mean ± S.E.M. *p < 0.05, **p < 0.01, ***p < 0.001.

Since we found an increase in the extracellular α-synuclein levels upon ambroxol treatment, and α-synuclein is known to be secreted via exosomes, we studied the possibility of ambroxol being able to induce exocytosis. BAF treatment was used as a positive control for exosomal release^30^. CD63 and flotillin, two proteins present in exosomes, were quantified in the extracellular media of neurons. Both 10 and 30 µM of ambroxol increased the levels of these exosome markers. The lower 10 µM dose of ambroxol led to a 44% increase of CD63 (p = 0.04) and a 24% increase in Flotilin1 (p = 0.03) whereas 30 µM of Ambroxol increased CD63 by 62% (p = 0.008) and Flotillin1 by 53% (p = 0.04) compared to control (Fig. 6A,B) We also analysed the levels of LAMP1, which is found in exosomes. We detected again that both doses of ambroxol increased the extracellular presence of LAMP1 in the extracellular neuronal media. The lower ambroxol dosage (10 µM) increased LAMP1 levels by 38% (p = 0.02), whereas 30 µM of ambroxol increased the release of LAMP1 by 172% (p = 0.03) (Fig. 6C). We also assessed the intracellular levels of Rab11, as it is a protein required for exocytosis and recycling endosomes. We found that the levels of Rab11 were increased with both doses compared to control (10 µM: 163%, p = 0.02; 30 µM: 178%, p = 0.02, Fig. 6D) which corroborates the hypothesis that ambroxol is inducing exocytosis.

### Effect of ambroxol on Gba1-deficient neurons

**Figure 7:**
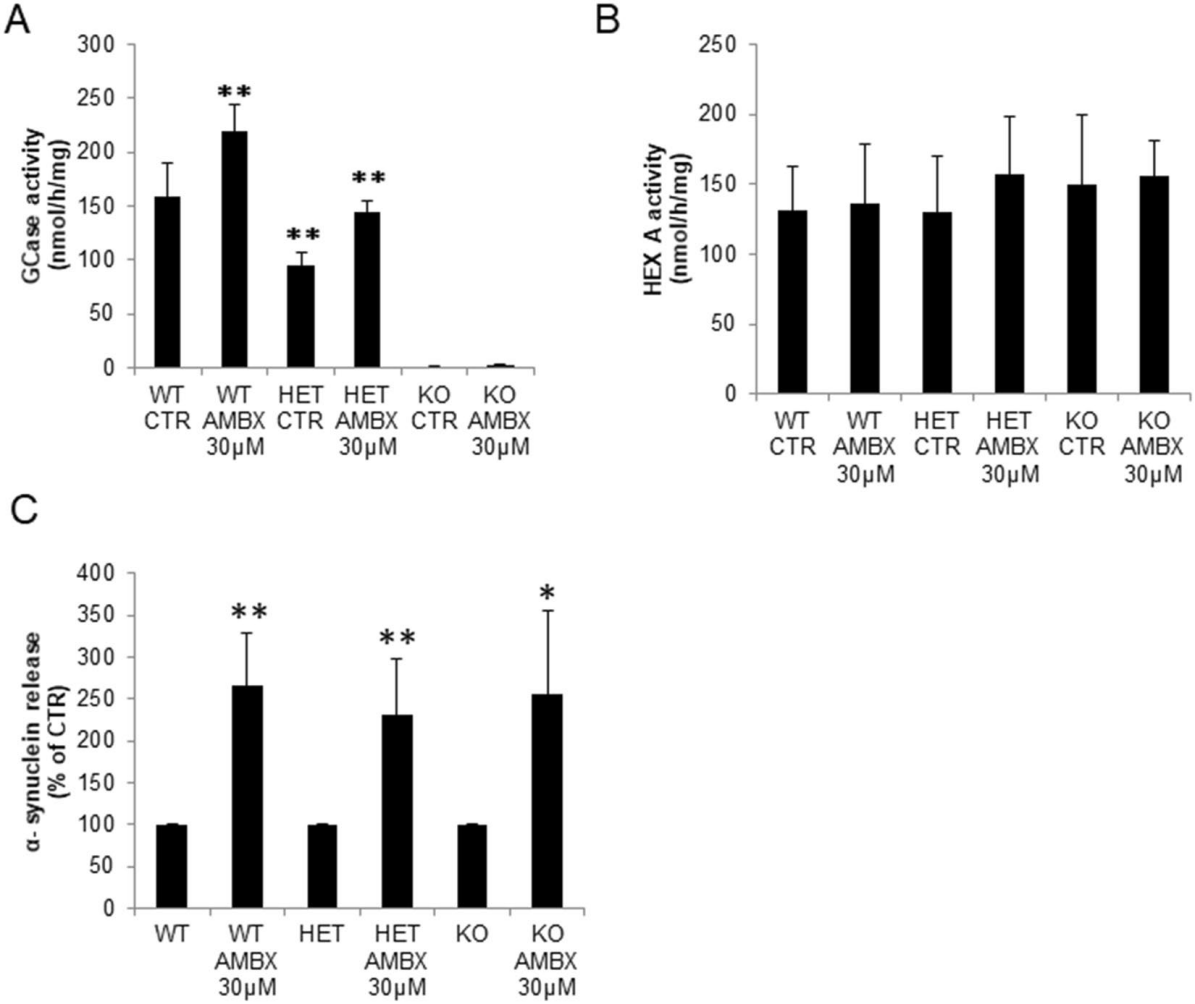
Ambroxol treatment of Gba1 deficient neurons. (**A)** GCase activity of WT and GBA HET neurons increased with ambroxol treatment, whereas GCase activity is unchanged in GBA KO treated with ambroxol (n = 5). (**B)** Hex A activity is not changed by ambroxol in any of the genotypes (WT, GBA HET or KO) (n = 4). **(C)** α-synuclein release was also increased following ambroxol treatment in WT, GBA HET and GBA KO neurons (n = 3). Data presented in % of CTR. All data represent mean ± S.E.M. *p < 0.05, **p < 0.01, ***p < 0.001.

To understand whether some of the effects of ambroxol are dependent on GCase expression and activity we assessed the effect on *Gba1*^*−/−*^ (GBA KO) and *Gba1*^*−/+*^ (GBA HET) primary cultures. GBA HET neurons showed a significant decrease in GCase activity (58% decrease, p = 0.003), whereas GBA KO neurons had negligible GCase activity (KO: 1.19 nmol/h/mg vs WT: 159 nmol/h/mg). Treatment with 30 µM of ambroxol increased WT neurons GCase activity by 37% (p = 0.004) and GBA HET neurons by 50% (p = 0.001) whereas no changes were observed in GBA KO (Fig. 7A). No differences were observed in Hex A activity in WT, HET or KO GBA neurons (Fig. 7B). Extracellular release of α-synuclein was also increased in GBA HET and GBA KO upon treatment with 30 µM ambroxol (Fig. 7D). Together our data indicate that some of the effects of ambroxol in neurons occur independently of GCase activity.

## Discussion

Small molecule chaperones that increase GCase activity are being studied for their potential to be disease-modifying therapies for PD with *GBA1* mutations^18–20,22,23,31–33^. Since GCase activity is also decreased in sporadic PD^8,24^ and ambroxol has been shown to increase WT GCase and other lysosomal proteins in control fibroblasts^18–20^ we have investigated the effect of ambroxol on mouse neurons containing WT GCase.

In a recent study from our laboratory WT mice were treated with ambroxol at a concentration of 4 mM in drinking water resulting in significantly increased brain GCase activity of 16–22%^23^. However, the amount of ambroxol that actually reaches the brain, and neurons in particular, is unknown. Human studies have reported ambroxol concentrations in blood or CSF of up to 3 μM, although this will be dependent on the dosing regimen used^34,35^. We report that 10 μM is sufficient to increase GCase activity in primary neurons. Previous reports in fibroblasts, SH-SY5Y and neural crest stem cell-derived dopaminergic neurons have used 60 µM of ambroxol^18,19,31^ but we found this dose to have cytotoxic effects on primary mouse cortical neurons.

Similar to what was reported in fibroblasts^18,19^ we have found that ambroxol increased *Gba1* mRNA, protein levels and activity, as well as, several lysosomal proteins such as cathepsin D, LAMP1 and the GCase transporter LIMP2. Nuclear translocation of TFEB, a master regulator of lysosomal biogenesis^21^ was increased upon treatment with ambroxol which probably contributes to the increased lysosomal content and number of acidic vesicles in these cells.

Activation of TFEB is commonly associated with activation of macroautophagy, particularly following starvation and inhibition of mTOR^28,36^, but is also increasingly associated with other physiological responses ranging from lysosomal exocytosis, lipid catabolism, ER stress and the immune response^26,37–40^. Macroautophagy flux appeared to be inhibited in ambroxol treated mouse cortical neurons as LC3-II, a marker of autophagosome number, was increased under basal levels but was not greatly increased in the presence of bafilomycin A1. This was not expected as ambroxol has been shown to increase macroautophagy in human neuronal cells containing *GBA1* mutations^31^. The lower dose or different type of neuron might account for the discrepancy. It should also be noted that mouse cortical neurons were treated at day 4 or 5 *in vitro*, whereas the human neurons were differentiated for several weeks. Autophagy inhibition appears to play a role in axon development and branching in developing mouse cortical neurons^41,42^, whereas lysosomal content increases significantly as cultured human neurons mature^9,31^. Either of these observations might explain the differing susceptibility of these neurons to ambroxol. The mechanism by which TFEB is activated following ambroxol treatment in mouse cortical neurons requires further investigation. For example, is it a compensatory mechanism due to the inhibition of macroautophagy following ambroxol treatment? Or perhaps a direct consequence of ambroxol treatment affecting the phosphorylation status of TFEB by activating/inactivating a kinase or phosphatase, and thus it’s propensity to translocate to the nucleus.

Given the observed increase in exocytosis following ambroxol treatment, perhaps primary mouse neurons at these lower doses respond by increasing the secretory pathway, rather than autophagy. Ambroxol is a mucolytic used primarily to treat respiratory diseases, where it acts as a secretory agent. Fois *et al*. have shown that ambroxol in concentrations greater than 1μM accumulates in lamellar bodies, which are the acidic Ca^2+^ stores in pneumocytes, much like lysosomes, leading to the release of Ca^2+^ and increased exocytosis^34^. Notably release of lysosomal Ca^2+^ activates calcineurin, which can then dephosphorylate and thus activate TFEB^43,44^. TFEB activation has been implicated in regulating lysosomal exocytosis^37,45^, by increasing the pool of lysosomes in proximity to the plasma membrane and promoting the fusion of lysosomes with the plasma membrane. In lysosomal storage diseases, lysosomal exocytosis has been described as a beneficial event that relieves the cells from storage material and degradation products^46,47^. Lysosomal exocytosis has also been linked to plasma membrane repair, neurite outgrowth, improved phagocytosis and the release of signalling molecules and digestive enzymes^46,48,49^. Lysosomal exocytosis has been reported to generate specific plasma membrane domains containing LC3-II^49^. If this occurs in neurons, it might contribute to the higher LC3-II levels observed following ambroxol treatment that were not increased further by bafilomycin A1 treatment.

The decrease in α-synuclein phosphorylated at Ser129 in neurons treated with ambroxol is similar to that observed in mice over expressing human a-synuclein treated with ambroxol^23^. It is unclear if this decrease is due to increased exocytosis of α-synuclein or via another mechanism.

The observation that *Gba1* KO neurons exhibit a similar response to ambroxol with regards to α-synuclein release as WT neurons indicates that this effect is independent of chaperoning GCase.

We proceeded to investigate the effect of ambroxol on PGC1-α levels as this protein is known to be regulated by TFEB^26,27^. PGC1-α has been described to be involved in mitochondria biogenesis^29^. We found that *Pgc1-α* mRNA levels were increased upon ambroxol treatment in neurons. Coincident with this, mitochondrial proteins were also found increased in neurons treated with ambroxol, pointing to an increase in mitochondria content in treated neurons. Mitochondrial defects have been found in cell and animal models of GCase deficiency^50–54^ and in idiopathic PD^55–57^. mTOR, a master regulator upstream of TFEB and PGC1-α, has also been found to be decreased in GCase deficiency models^11,58^. It is interesting to speculate that ambroxol’s effects on TFEB and PGC1-α would be beneficial to restore both mitochondrial function and rebalance lysosomal metabolism in PD.

In summary, our data suggest that ambroxol, has several actions beyond its chaperone activity; it influences lysosomal and mitochondrial function, and increases protein exosomal release. Its ability to reduce cellular phosphorylated α-synuclein in *in vitro* and *in vivo* models highlights its potential application to PD to modify the progression of this disease. Clinical trials are currently underway to test this hypothesis.

## Methods

### Ethical Approval

The *Gba1* knockout mouse model and wild type (WT) colony was covered by project licence 70/7685 issued by the United Kingdom Home Office. All animal procedures were carried out as described in the above project licence and the United Kingdom Animals (Scientific Procedures) Act of 1986 (Schedule 1). All efforts were made to reduce the number of animals by following the 3 R’s.

### Primary Cortical Mouse Neurons

WT mice and Heterozygotic *Gba1* transgenic mice in which a loxp-neo-loxp (lnl) cassette was inserted into exon 8 of one *Gba1* allele in all cell types^59^ were used to generate mouse embryonic primary cortical neurons as follows. Cortex from day 15 embryos were dissected, meninges removed, homogenized by passing through a 23 G needle and centrifuged at 1000 rpm for 5 min. Cell pellets were resuspended in neuronal media which consisted of neurobasal media (Invitrogen) supplemented with B27 (Invitrogen), glutamax (Sigma) and antimycotic/antibiotic solution (Sigma) and seeded onto poly-ornithine coated plates (Sigma). Neurons were left in culture for no longer than 15 days.

### Ambroxol treatment of cortical neurons

Ambroxol hydrochloride (Sigma) was prepared in DMSO at a concentration of 10 mM, in order to be administered to neurons in culture at a concentration of 10, 30 and 60 µM. At day 4 or 5 in culture neuronal media was changed to fresh neuronal media with the different doses of ambroxol. 3 days after the first treatment with ambroxol, the neurons were treated again for an extra 2 days. After 5 days of ambroxol treatment neurons were harvested and assays performed.

### Western blotting

Neurons were harvested and lysed in RIPA buffer supplemented with protease and phosphatase inhibitors. Nuclei and cell debris were removed by centrifugation at 14000 rpm for 10 min. For intracellular detection of total α-synuclein levels neurons were lysed in 0.1% SDS, 150 mM NaCl, 10 mM Tris, pH7.5 supplemented with RQ1 DNase (Promega) and protease and phosphatase inhibitors and incubated at 37 °C for 1 hour. Protein (5–30 µg) was separated on 4–12% or 12% Bis-Tris NuPAGE gels (Invitrogen) and transferred to Hybond P membrane (GE Healthcare, Little Chalfont, UK). Blots were incubated with antibodies against Cathepsin D (CTD-19, 1:2000, abcam, Cambridge, UK), LAMP1 (1:1000 Abcam), LAMP2a (1:1000 Abcam) Hsc70 (EP1531Y, 1:500 Abcam), LC3B (1:500, Abcam), SQSTM1/p62 (1:1000, Abcam), α-synuclein (4D6,1:1000; Abcam), phospho (S129) α-synuclein (1:500, Abcam), Lamin A (1:500, Abcam), LIMP2 (1:2000, Abcam), COXIV (1:1000, Abcam), Prohibitin1 (1:1000, Abcam), Rab 11 (BD transduction laboratories, 1:1000), TFEB (1:1000, Abcam)or β-actin (1:5000, Abcam). Bands were detected with respective horse radish peroxidase-linked secondary antibodies (Dako, Glostrup, Denmark) and enhance chemiluminescence (Luminata Forte, Merck Millipore). Density of bands was determined using Image Lab software (BioRad).

### Nuclear extracts

To prepare nuclear extracts of neurons treated with ambroxol we used the EpiQuik Nuclear Extraction kit (Epigentek), as per manufacturer’s instructions. Then, western blotting was performed on the cytoplasmic and nuclear fractions.

### Dot blotting

We used the Bio-Dot SF blotting apparatus from Bio-Rad to analyse media collected from neuronal cultures treated with ambroxol for 5 days. Before loading the media in the apparatus, debris and floating cells were removed by centrifugation. Bio-Dot SF blotting apparatus was assembled following the instruction manual and 100μl of media was loaded in the respective well. Dot blots were then washed with PBS and then blocked with Block Ace (BioRad) ON at 4 °C, followed by incubation with antibodies against CD63 (Santa Cruz Biotechnology, 1:100), Flotillin1 (Abcam, 1:1000), Lamp1 (Abcam 1:1000).

### Lysosomal Enzyme Assays

Cell lysates were prepared as above, sonicated and GCase activity determined in samples (20 µg protein) by hydrolysis of 5 mM 4-methylumbelliferyl-β-D-glucopyransoside in McIIvaine buffer (pH 5.4) in the presence of 22 mM sodium taurocholate at 37 °C for 1 hour. The reaction was stopped by addition of 0.25 M glycine (pH 10.4) and 4-methylumbelliferone fluorescence measured at excitation 360 nm, emission 460 nm.

β-hexosaminidase A was assayed in lysates (2 µg protein) using the fluorogenic substrate 4-methylumbelliferyl-N-acetyl-glucosaminide (2 mM), in sodium citrate buffer (pH 4.2) at 37 °C for 30 minutes^60,61^. The reaction was stopped by addition of 0.25 M glycine (pH 10.4) and fluorescence measured as above.

### Lysosomal function: Lyso-ID Green Detection Kit

Acidic vesicles were detected in live cells using the Lyso-ID Green detection kit (Enzo Lifesciences, Farmingdale, NY, USA). Cells were seeded in 12 well plates (90% confluent) and incubated with 4 µl/ml Lyso-ID in assay buffer at 37 °C for 30 minutes. Cells were washed with assay buffer and fluorescence measured on a plate reader at excitation 488 nm, emission 520 nm. Following reading, buffer was aspirated, cells lysed overnight in 0.25 M NaOH and protein concentration measured. Cell fluorescence was expressed as fluorescent units/mg protein.

### Quantitative Real-Time PCR

RNA was extracted from neuronal cells using RNeasy kit (Qiagen) and converted to cDNA using nanoScript2 RT kit (PrimerDesign). qPCR was performed in a STEP One PCR machine (Applied Biosystems) using the Power SYBR Green PCR master mix (Applied Biosystems). Primer used are listed in Table 1. β-Actin mRNA levels were used to normalize the data. Relative expression was calculated using the ΔCT method.

**Table 1:** List of primers used for qPCR analysis.

Target	Sequence
α-synuclein	5′-CAGAGGCAGCTGGAAAGACA- 3′ 5′- CACCACTGCTCCTCCAACAT-3′
Gba1	5′-GACCAACGCTTGCTGCTAC-3′ 5′- ACAGCAATGCCATGAACGTA-3′
Cathepsin D	5′-CCCTCCATTCATTGCAAGATAC-3′ 5′-TGCTGGACTTGTCACTGTTGT-3′
PGC1α	5′-AGCCGTGACCACTGACAACGAG-3′ 5′-GCTGCATGGTTCTGAGTGCTAAG-3′
COX 3	5′- CGTGAAGGAACCTACCAAGG-3′ 5′-CGCTCAGAAGAATCCTGCAA-3′
mt-ND6	5′-TGTTGGAGTTATGTTGGAAGGAG-3′ 5′-CAAAGATCACCCAGCTACTACC-3′

### Measurement of α-synuclein by ELISA

Conditioned media was removed from neurons at day 5 of ambroxol treatment and debris/floating cells removed by centrifugation. Neurons were harvested and cell pellets frozen. The amount of α-synuclein released in to media was measured by ELISA (Sensolyte; AnaSpec) as per manufacturer’s instructions. Cell pellets were lysed and protein content calculated using BCA protein assay kit (ThermoScientific). Data were expressed as pg α-synuclein/mg protein.

### Statistical analysis

Statistical analyses were performed by Student’s t test. Values with p < 0.05 were considered significant.

## Electronic supplementary material

Supplementary Information
