# Supplementary material for: Effects of ambroxol on the autophagy-lysosome pathway and mitochondria in primary cortical neurons
Source: Sci Rep. 2018 Jan 23;8:1385. doi: 10.1038/s41598-018-19479-8 (PMC5780491; doi:10.1038/s41598-018-19479-8)
Supplement: Supplementary file 1 — Supplementary Information [file 41598_2018_19479_MOESM1_ESM.pdf]

Effects of ambroxol on the autophagy-lysosome pathway and mitochondria in primary cortical neurons

Magalhaes J[1], Gegg ME[1], Migdalska-Richards A[1], Schapira AH[1*]

[1]Department of Clinical Neuroscience, Institute of Neurology, University College London, London NW3 2PF, UK

*Correspondence to: Professor A.H.V. Schapira, Chairman and Head of Department, Department of Clinical Neurosciences, Upper level 3, UCL Institute of Neurology, UCL, Rowland Hill Street, London NW3 2PF, UK.

E-mail: a.schapira@ucl.ac.uk; Tel: 020 7830 2012 Fax: 02074726829

Supplementary information

Methods

Cell viability assay: Live/Dead assay

To measure neuronal viability upon ambroxol treatment we used the LIVE/DEAD Viability/Cytotoxicity Kit (Thermofisher scientific). Briefly, we plated neurons in glass bottom dishes, treated with different concentrations of ambroxol. After 5 days of treatment cells were stained with 2 µM of Calcein AM and 4 µM of Ethidium homodimer-1 for 15 min at RT. Stained neurons were then visualised under a Nikon Eclipse Ti-E inverted microscope system using a 60x/1.4 oil objective and a Hamamatsu ORCA- Flash 4.0 camera. Images were acquired using NIS Elements AR software.

Measuring Neurite Length

Neurons were cultured on coverslips and treated with ambroxol as described above. Neurons were fixed in 3.7% paraformaldehyde (pH 7.4), permeabilised with methanol for 10min at -20°C, blocked in 2% (v/v) goat serum in PBS with 0.1 % (v/v) Triton X-100 for 1h at RT, and then incubated with β-Tubullin III antibody (Abcam, 1:500) for 1h at 37 °C. Following washing of coverslips with PBS, cells were incubated with anti-rabbit alexa fluor (Invitrogen) for 1h at 37 °C, washed with PBS and coverslips mounted in citifluor containing DAPI. Images were obtained with a Nikon Eclipse Ti-E inverted microscope system using a 60x/1.4 oil objective, a Hamamatsu ORCA- Flash 4.0 camera and the NIS Elements AR software. Image J was used to measure the neurite length.

Preparation of Soluble/Insoluble cellular fractions

Neurons were homogenised in high salt buffer (50mM Tris pH7.5, 750mM NaCl, 5mM EDTA, 1% TritonX-100, DNase and protease inhibitors). Samples were incubated 15 min at RT, sonicated for 1 min and centrifuged at 14000rpm for 10 min. Supernatant was collected (soluble fraction) and the pellet was homogenised in Urea-SDS buffer (8M Urea, 2%SDS,

10mM Tris pH 7.5, DNase and protease inhibitors). Samples were incubated for 15 min at RT and centrifuged at 14000rpm for 10 min. Supernatant was collected (insoluble fraction). Western blotting was performed for the soluble and insoluble fraction.

Supplementary Figures

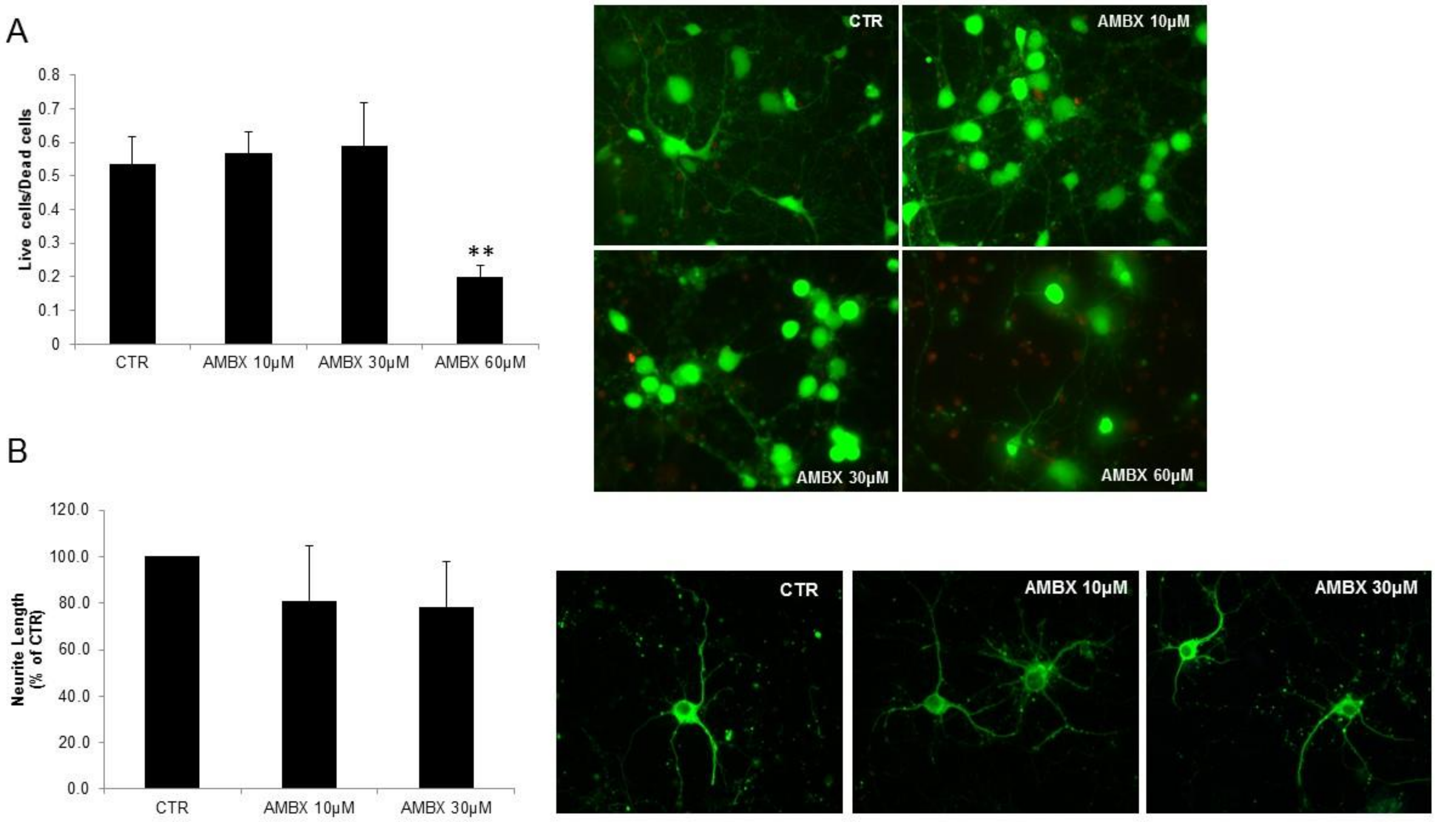

Figure SI 1. *Optimization of ambroxol concentration*. **A.** Neuronal viability measured in number of dead cells. 60µM of ambroxol increased the number of dead cells whereas 10µM and 30µM doses did not induced any change compared to control (n=3) **B.** Both 10µM and 30µM of ambroxol did not change the neurite length compared to control neurons (n=3). Data presented in % of CTR. All data represent mean±S.E.M. $p^{*}<0.05$, $p^{**}<0.01$, $p^{***}<0.001$.

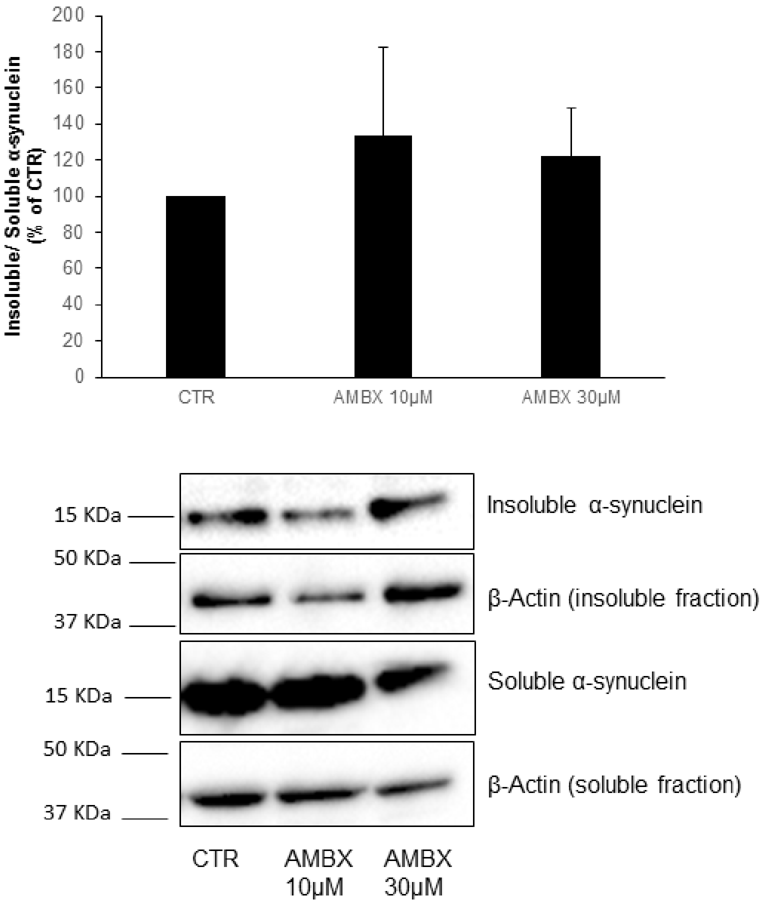

Figure SI 2. *Effect of ambroxol on insoluble species.* The level of Insoluble species in neurons was not altered by the treatment with ambroxol (n=2). Data presented is % of CTR. Blots have been cropped. All data represent mean±S.E.M. p*<0.05, p**<0.01, p***<0.001.

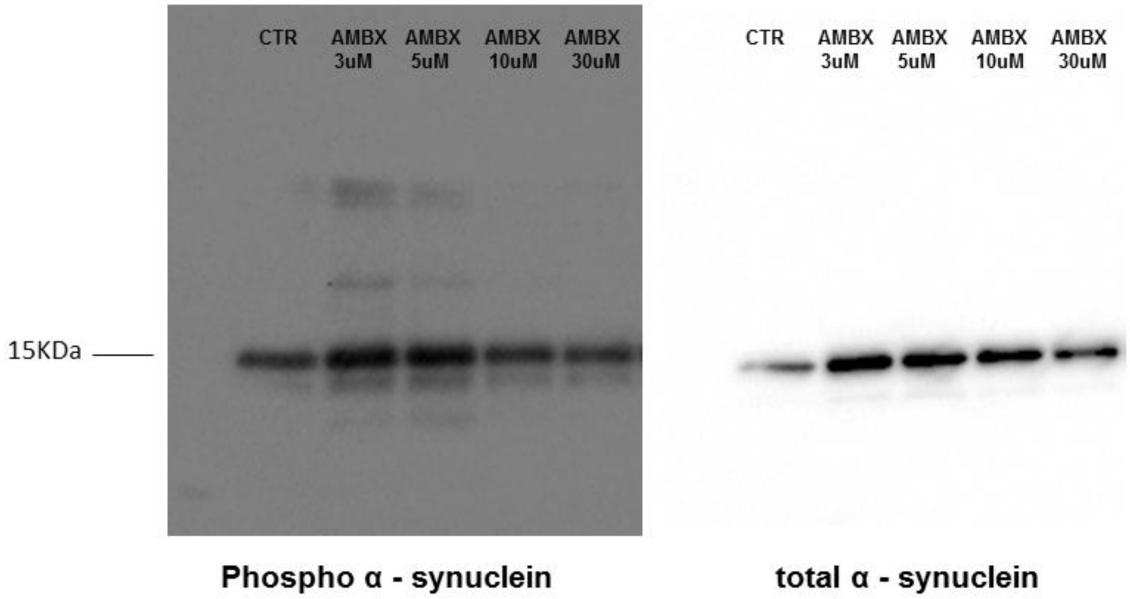

Figure SI 3. *Full length blot from Figure 5D.*
